# Overview of Chemotherapy for Gastric Cancer

**DOI:** 10.3390/jcm12041336

**Published:** 2023-02-07

**Authors:** Yasushi Sato, Koichi Okamoto, Yoshifumi Kida, Yasuhiro Mitsui, Yutaka Kawano, Masahiro Sogabe, Hiroshi Miyamoto, Tetsuji Takayama

**Affiliations:** 1 Department of Community Medicine for Gastroenterology and Oncology, Tokushima University Graduate School of Medical Science, Tokushima 770-8503, Japan; 2 Department of Gastroenterology and Oncology, Tokushima University Graduate School of Medical Science, Tokushima 770-8503, Japan

**Keywords:** gastric cancer, biomarker, HER2, ICI

## Abstract

Gastric cancer (GC) is one of the most clinically challenging cancers worldwide. Over the past few years, new molecular-targeted agents and immunotherapy have markedly improved GC prognosis. Human epidermal growth factor receptor 2 (HER2) expression is a key biomarker in first-line chemotherapy for unresectable advanced GC. Further, the addition of trastuzumab to cytotoxic chemotherapy has extended the overall survival of patients with HER2-positive advanced GC. In HER2-negative GC, the combination of nivolumab, an immune checkpoint inhibitor, and a cytotoxic agent has been demonstrated to prolong the overall survival of GC patients. Ramucirumab and trifluridine/tipiracil, which are second- and third-line treatments for GC, and trastuzumab deruxtecan, an antibody–drug conjugate for HER2-positive GC, have been introduced in clinics. New promising molecular-targeted agents are also being developed, and combination therapy comprising immunotherapy and molecular-targeted agents is expected. As the number of available drugs increases, it is important to understand the target biomarkers and drug characteristics and select the optimal therapy for each patient. For resectable disease, differences in the extent of standard lymphadenectomy between Eastern and Western countries have led to different standard treatments: perioperative (neoadjuvant) and adjuvant therapy. This review aimed to summarize recent advances in chemotherapy for advanced GC.

## 1. Introduction

In 2018, more than one million new cases of GC were diagnosed, and more than 782,000 deaths were recorded worldwide [1]. The highest mortality rates from GC have been reported in East Asia, including Japan, Korea, and China [2], and the lowest rates have been reported in North America. The mortality rate is mainly dependent on the rate of *Helicobacter pylori* (HP) infection, as HP infection is a known dominant cause of GC [3]. Owing to the improved treatment of HP, the rate of GC has decreased [4,5]. However, the proportion of proximal GCs, which often lead to a poor prognosis, is increasing [6]. The 5-year survival rate for GC is 60% or higher in Japan [7] and Korea [8], where more than half of GC patients are diagnosed at an early stage through well-organized population screening programs [9,10]. In contrast, GC is often diagnosed at an advanced stage in most Western patients. Notably, the biological nature of GC and differences in therapeutic quality between Eastern and Western countries may affect treatment outcomes [11,12,13].

In terms of the management of localized operable disease, marked disparities between the East and West regarding surgical procedures [14] and their outcomes result in sizeable geographical variation in the preferred adjuvant treatment for localized GC [15]. For example, adjuvant chemoradiotherapy (CRT) is commonly used following D0/1 surgery for patients with GC in the US [16], and an approach with intensive combination chemotherapy administered before and after surgery (perioperative) is preferable in the UK [17]. In East Asia, adjuvant oral fluoropyrimidine-based chemotherapy following D2 resection is considered the gold standard [18].

In terms of the treatment for metastatic and unresectable GC, the first-line systemic chemotherapy for metastatic disease recommended by international consensus groups has been a combination of fluoropyridine and platinum chemotherapy, in which patients with metastatic GC have a median overall survival (OS) of approximately 1 year (Asian patients survive longer), for the past several decades [19]. Owing to achievements in chemotherapy and targeted therapy [20], the mortality rate has gradually decreased in recent decades. In 2010, the combination of human epidermal growth factor receptor 2 (HER2)-directed trastuzumab and chemotherapy was reported to improve the survival prognosis of patients with gastric and gastroesophageal cancers [21]. However, even with such chemotherapy and molecular-targeted therapy, the median OS is difficult to exceed 2 years, and the 5-year OS rate for advanced GC ranges from 10 to 15% [19,22]. 

These poor results can be attributed to the fact that until recently, only HER2 positivity was established as a drug-treatable target [22]. More importantly, as treatment resistance is largely attributable to the heterogeneity of GC, a better understanding of its molecular biology and new personalized therapeutic approaches is expected [23].

Although targeted therapies and chemotherapy primarily attack cancer cells directly, clinical trials are currently focused on immune checkpoint inhibitors (ICIs), which include anti-programmed death-1 (PD-1), anti-PD-L1, and anti-cytotoxic T lymphocyte antigen 4 (CTLA-4). ICIs are increasingly being administered to patients with late-stage malignant tumors who have failed multiple treatments, such as melanoma [24], non-small cell lung cancer [25], and colorectal cancer [26], and have achieved deep and durable tumor responses. Regarding GC, ICI has emerged as a promising therapeutic agent, and, to date, nivolumab and pembrolizumab have been recommended as second-line or subsequent therapies in GC guidelines globally [27,28,29]. Nivolumab and chemotherapy have been recognized as first-line treatments based on the results of two global phase 3 studies [30,31,32]. Moreover, some biomarkers, including microsatellite instability-high (MSI-H) or PD-L1, have been demonstrated to be potential predictors of the outcomes of GC patients receiving ICI [33]. 

GC treatment has been heterogeneous worldwide owing to differences in morbidity, mortality, and medical resources. However, as treatment selection by biomarkers has progressed in recent years, there has been an increasing trend toward the use of common regimens in many countries. As HER2 is currently the only established therapeutic target, this review sought to provide a comprehensive overview of first-line therapy, second-line therapy, and subsequent palliative systemic therapy for metastatic GC in HER2-positive and HER2-negative GC from the pivotal clinical trials reported by January 2023 and present recent evidence for perioperative (neoadjuvant) and adjuvant therapy.

## 2. First-Line HER2-Negative Gastric Cancer 

### 2.1. Doublet Chemotherapy

The Asian, US, and European guidelines recommend a regimen of oral or intravenous injection of fluoropyrimidine combined with platinum as the palliative first-line chemotherapy for HER2-negative GC [27,28,29]. Pivotal randomized controlled clinical trials for first-line HER2-negative GC are listed in Table 1. The efficacy of cisplatin + S-1 (SP) or capecitabine (XP) doublet combination therapy has been demonstrated by the results of several phase III trials, including the SPIRITS study and JCOG 9912 study [21,34,35]. Among the platinum agents, cisplatin has been mainly used; however, its strong gastrointestinal toxicity, nephrotoxicity, and other side effects have become problematic; thus, other platinum agents have been investigated. In particular, SOX (S-1 + oxaliplatin) and XELOX (oxaliplatin + capecitabine) regimens containing oxaliplatin are recommended as they are easier to administer than SP and XP and do not require hydration. This recommendation is based on evidence obtained from the REAL-2 trial, which revealed that capecitabine and oxaliplatin are as effective as fluorouracil and cisplatin [36]. In fact, the G-SOX study demonstrated that SOX is as effective as FP and has a favorable safety profile [37]. 

Interestingly, in the GO2 phase III trial, the XELOX regimen with a 60% dose reduction from the standard dose was demonstrated to be less toxic and non-inferior in terms of progression-free survival (PFS) in elderly and frail patients [38]. In addition, the combination of 5-FU/levofolinate (LV) and oxaliplatin (FOLFOX) has demonstrated efficacy and is particularly useful in patients with intestinal obstruction or other difficulties with oral intake [39,40]. S-1 plus docetaxel is conditionally recommended in Japan, as the START trial highlighted its OS superiority over S-1 monotherapy in patients who cannot receive platinum-containing regimens [41]. 

### 2.2. Triple Chemotherapy

There are divided views on the three-drug combination therapy. A phase III V325 trial in the U.S. and Europe revealed the superiority of docetaxel/cisplatin/5-fluorouracil (DCF) triple therapy, in which docetaxel was added to 5FU plus cisplatin infusion [42]. However, this three-drug combination was not recommended in Asian guidelines owing to its high toxicity, which was not balanced with efficacy and was only recommended for a small number of tolerable patients. Recently, in Japan, the triple combination of docetaxel, cisplatin, and S-1 (DCS) was compared to SP in a phase III trial (JCOG1013) following the results of a promising phase II trial [43,44]; however, no OS benefit was found [45]. As a result, triple-drug combination therapy, including taxanes, is not currently recommended as a first-line therapy in Japan. There are many criticisms that the study did not employ an appropriate dosage regimen and ultimately did not achieve satisfactory results. Several studies have reported the usefulness of triple-drug combination therapy in cases where conversion therapy is intended [46,47].

### 2.3. Immunotherapy

In recent years, ICIs have been introduced as a new standard of care for several malignancies, including advanced GC, and have demonstrated good clinical efficacy [48]. To date, the KEYNOTE-062, ATTRACTION-4, and CheckMate 649 trials serve as the major trials that used ICI as the primary treatment for GC. In the KEYNOTE-062 trial, 763 patients (69% with GC) were randomized to receive pembrolizumab alone, pembrolizumab plus chemotherapy (cisplatin/5-FU or capecitabine), and chemotherapy plus placebo as first-line treatment for HER2-negative and PD-Ll-positive (combined positive score (CPS) ≥ 1) unresectable advanced or recurrent GC and esophagogastric junction cancer [49]. In patients with PD-L1 CPS ≥ 1, pembrolizumab monotherapy was non-inferior to chemotherapy (median OS 10.6 vs. 11.1 months (HR, 0.91; 99.2% CI, 0.69–1.18; noninferiority margin, 1.2)) but prolonged OS vs. chemotherapy in patients with CPS ≥ 10 (median OS 17.4 vs. 10.8 months, HR 0.69 (95% CI 0.49–0.97)). In particular, in the group of patients with PD-L1 CPS ≥ 1 and MSI-high tumors, the survival benefit was enhanced with pembrolizumab (HR, 0.29; 95% CI, 0.11–0.81) and pembrolizumab plus chemotherapy (HR, 0.37; 95% CI, 0.14–0.97) vs. chemotherapy, demonstrating the benefit of pembrolizumab in MSI-H tumors [49].

ATTRACTION-4 is a phase II/III trial conducted in Japan, Korea, and Taiwan [30]. In the open-label section, the superiority of nivolumab plus chemotherapy (SOX or oxaliplatin + capecitabine [CapeOX]) over chemotherapy as a first-line treatment for unresectable advanced or recurrent GC and esophagogastric junction cancer was verified. This study revealed the superiority of nivolumab plus chemotherapy (SOX or CapeOX) over chemotherapy as a first-line treatment and showed a significant increase in PFS (median PFS: 10.45 months vs. 8.34 months, HR: 0.68, 98.51% CI: 0.51–0.90, *p* = 0.0007), which was one of the primary endpoints. However, no significant difference was found for the other primary endpoint, OS (median OS: 17.45 months vs. 17.15 months, HR: 0.90, 95% CI: 0.75–1.08, *p* = 0.257), which is presumably because many patients received subsequent treatments or additional immunotherapy. The CheckMate 649 trial, a large, randomized, phase III study of 1,581 patients (24% Asian, 76% non-Asian, 100% adenocarcinoma) was conducted worldwide to analyze the superiority of nivolumab plus chemotherapy over chemotherapy or nivolumab plus ipilimumab [31,32]. Sixty percent (n = 955) of these patients had a PD-L1 CPS score ≥ 5. The primary endpoints of OS (median OS 14.4 vs. 11.1 months (HR 0.71 (98.4% CI (0.59–0.86)), *p* < 0.0001) and PFS (median PFS: 7.7 months vs. 6.0 months, HR: 0.68, 98% CI: 0.56–0.85, *p* < 0.0001) revealed the superiority of nivolumab plus chemotherapy. In addition, for OS, a statistically significant difference was found between CPS ≥ 1 patients (median OS: 14.0 months vs. 11.3 months, HR: 0.77, 99.3% CI: 0.64–0.92, *p* = 0.0001) and the overall population (median OS: 13.8 months vs. 11.6 months, HR: 0.80,99.3% CI: 0·68–0·94, *p* = 0·0002).

The PFS results also highlighted the superiority of nivolumab plus chemotherapy for CPS ≥ 1 cases and the overall population. The overall response rate (ORR) of patients with a CPS ≥ 5 was 60% vs. 45% (*p* < 0.0001), with a significantly higher value obtained in the nivolumab plus chemotherapy arm. Furthermore, an improved ORR was achieved in the nivolumab plus chemotherapy arm across all CPS subgroups, with patients with PD-L1 CPS ≥ 5 and MSI-H achieving a benefit, especially when administered the combination with immunotherapy. The combination of nivolumab and ipilimumab without chemotherapy had no clear OS benefit compared to chemotherapy alone. Based on these results, nivolumab plus chemotherapy in patients with advanced gastric, gastroesophageal junction, and esophageal adenocarcinoma, regardless of PD-L1 CPS status, was approved in the US, Taiwan, and Japan. In Europe, nivolumab plus chemotherapy has been approved for patients with a PD-L1 CPS of ≥ 5.

## 3. First-Line HER2-Positive Gastric Cancer

### 3.1. HER2-Targeted Therapy

HER2, also called ERBB2, is a receptor tyrosine protein kinase that is involved in cell proliferation through signaling pathways, such as RAS/RAF/MEK/ERK, PI3K/AKT/mTOR pathways, etc. [50]. HER2-positive tumors show amplification of the HER2 gene, which is commonly associated with protein overexpression, leading to tumorigenesis in GC [51]. Although only 15–20% of GC cases are HER2-positive [52], the clinical efficacy of trastuzumab, a HER2-targeted therapy, has been established for HER2-positive advanced GC. Trastuzumab is a monoclonal antibody that binds to the extracellular domain of the HER2 receptor and inhibits the HER2 signaling pathway. The pivotal randomized controlled clinical trials for first-line HER2-positive GC are listed in Table 2. The multicenter phase III ToGA trial revealed that trastuzumab plus cisplatin and fluoropyrimidine significantly improved the survival of patients with HER2-positive advanced gastric and gastroesophageal junction adenocarcinoma compared with that of patients treated with chemotherapy alone (median OS 13.8 vs. 11.1 months, HR 0.74, 95% CI 0.0–0.91; *p* = 0.0046). In a post-hoc subgroup analysis, the HER-2 overexpression (IHC3+ or IHC2+ and FISH-positive) showed an improvement in OS (median OS 16.0 vs. 11.8 months, HR 0.65, 95% CI 0.51–0.83; *p* = 0.036) [21].

Following the success of the ToGA trial, several randomized phase III trials evaluated the efficacy of other HER2-targeted therapies in patients with HER2-positive advanced gastric and gastroesophageal junction cancers [23,53]: first-line treatment with lapatinib + capecitabine + oxaliplatin (TRIO-013/LOGiC) [54], pertuzumab and trastuzumab + fluoropyrimidine + cisplatin (JACOB) [55], second-line treatment with lapatinib + paclitaxel (Tytan) [56], and T-DM1 (GATSBY) [57]. However, none of these treatments could improve the clinical outcomes of patients with HER2-positive GC. Several mechanisms have been proposed to cause resistance to HER2-targeted therapies, including (1) the intratumor heterogeneity of HER2, (2) aberrant activation of the PIK3CA signaling pathway (a downstream signal of HER2), and (3) simultaneous amplification of *EGFR*, *MET*, and *CCNE1* [23].

Several novel HER2-targeted drugs for GC are under development, including bispecific antibodies (zanidatamab) [58], antibody–drug conjugates (trastuzumab deruxtecan) [59], and small-molecule kinase inhibitors (afatinib, neratinib, and tucatinib) [60,61,62], which have been designed to overcome this resistance [63]. 

### 3.2. Anti-PD-1 Antibody Plus HER2-Targeted Therapy

In a HER2-positive immunocompetent mouse model, anti-PD-1 antibodies have been reported to significantly improve the antitumor activity of trastuzumab by enhancing antibody-dependent cellular cytotoxicity (ADCC) [64]. A phase II trial evaluating the efficacy of trastuzumab plus pembrolizumab in combination with chemotherapy revealed very promising results, with a median PFS of 13.0 months (95% CI, 8.6–NA) and median OS of 27.2 months (95% CI 18.8–NA) [65]. The phase III KEYNOTE-811 trial of pembrolizumab plus trastuzumab and chemotherapy revealed a statistically significant increase of 22.7% in the ORR in the pembrolizumab group compared to the placebo group (77.4% vs. 51.9%, *p* = 0.00006) [66]. The pembrolizumab group also displayed more profound responses than the placebo group (median change from baseline: 65% vs. 49%; 80% or ≥80% decrease from baseline: 32.3% vs. 14.8%). Further, more complete responses were observed in the pembrolizumab group than in the placebo group (11.3% vs. 3.1%). These interim results for KEYNOTE-811 led to the expedited FDA approval of the addition of pembrolizumab to trastuzumab and chemotherapy as the first-line treatment of patients with HER2-positive advanced GC. The KEYNOTE-811 trial demonstrated that the addition of ICIs to molecularly targeted therapy may be another potentially efficient strategy to overcome HER2 resistance in GC [66].

## 4. Second-Line and Subsequent Chemotherapy

The pivotal randomized controlled clinical trials for second-line and subsequent chemotherapy for GC are listed in Table 3. Several studies have shown that the administration of taxane or irinotecan results in higher survival rates than best supportive care as a second-line chemotherapy for GC patients with adequate performance status [67,68,69,70]. In addition, the efficacy of the anti-vascular endothelial growth factor receptor (VEGFR)-2 monoclonal antibody, ramucirumab, was proven in the REGARD and RAINBOW randomized phase III trials [71,72]. In the multicenter randomized phase III REGARD trial [72], patients with advanced gastric and gastroesophageal junction cancers who progressed after first-line chemotherapy were randomized to receive ramucirumab or a placebo. The median OS was 5.2 months for ramucirumab and 3.8 months for the placebo (*p* = 0.047). The randomized phase III RAINBOW trial evaluated paclitaxel with or without ramucirumab in patients with advanced gastric and gastroesophageal junction cancers who had progressed after primary chemotherapy [71]. Patients who received ramucirumab plus paclitaxel had significantly longer median OS (9.63 months) than patients receiving paclitaxel alone (7.36 months, *p* < 0.0001); the median PFS was 4.4 and 2.86 months, respectively; and the ORR was 6% for paclitaxel alone versus 28% for ramucirumab plus paclitaxel (*p* = 0.0001).

The KEYNOTE-061 trial comparing pembrolizumab alone with paclitaxel failed to meet its primary endpoint (superior OS for patients with PD-L1 CPS ≥ 1) for patients with advanced gastric and gastroesophageal junction cancers who progressed after first-line chemotherapy. However, pembrolizumab-treated patients with PD-L1 CPS ≥ 10 had superior survival rates compared to patients with PD-L1 CPS ≥ 1 [73]. Paclitaxel plus ramucirumab is generally recommended based on the results of the RAINBOW study and is currently administered in many cases. As pembrolizumab is highly effective in patients with MSI-H, pembrolizumab must be used appropriately via the performance of the MSI test [74].

Recently, several new drugs have been developed as treatment options for ineffective secondary chemotherapy. The ATTRACTION-2 trial, a phase III study that sought to evaluate the efficacy of nivolumab in GC after third-line treatment [75], revealed the superiority of nivolumab over a placebo in OS (nivolumab vs. placebo, median 5.26 months vs. 4.14 months, HR 0.62, 95%CI 0.50–0.75). Notably, the response rate to nivolumab was 11.9%, but the OS in responders was very long (26.68 months). However, the effectiveness of nivolumab in combination with chemotherapy as the first-line treatment for HER2-negative GC has been demonstrated, which may reduce the possibility of using nivolumab as a third-line therapy. A phase III study (TAGS study) was conducted to determine the efficacy of trifluridine/tipiracil (FTD/TPI) as a third-line or later drug and revealed the superiority of FTD/TPI over a placebo in terms of OS (FTD/TPI vs. placebo, median 5.7 months vs. 3.6 months, HR 0.69, 95% CI 0.56–0.85) [76]. Of note, grade 3 or higher neutropenia was observed in 34% of patients, whereas febrile neutropenia was only observed in 2% of patients. FTD/TPI is an oral drug. As many patients with GC have poor oral intake, it is important to administer FTD/TPI when oral intake is still possible.

As mentioned above, no drug has demonstrated efficacy in HER2-targeted therapy for GC, except trastuzumab, as a first-line therapy. A phase II study (DESTINY-Gastric01 study) was conducted to explore the efficacy of trastuzumab deruxtecan (T-DXd) in HER2-positive GC. T-DXd displayed efficacy for the first time as a HER2-targeted therapy after second-line treatment. The DESTINY-Gastric01 trial randomized patients 2:1 to receive T-DXd or chemotherapy [77]. The efficacy of T-DXd was very high, with an ORR of 51% for T-DXd and 14% for chemotherapy (*p* < 0.001). Further, 9% of patients (11/119 patients) had complete response to T-DXd. The OS and PFS results were also promising with T-DXd (T-DXd vs. chemotherapy, median OS 12.5 vs. 8.4 months, HR 0.59, 95% CI 0.39–0.88; median PFS 5.6 vs. 3.5 months, HR 0.47, 95% CI 0.31–0.71). The most frequent grade 3 or higher adverse events were neutropenia (51%), anemia (38%), leukopenia (21%), and anorexia (17%). In addition, febrile neutropenia was observed in six patients and drug-induced pneumonia was observed in 10% (12/125) of patients. Although T-DXd-related interstitial lung disease is an adverse event requiring caution, it is considered a manageable adverse event with nine cases of grade 1/2, two cases of grade 3, and one case of grade 4 recorded. T-DXd is preferred over nivolumab as a third-line therapy for HER2-positive patients owing to its high therapeutic efficacy.

## 5. Adjuvant Therapy for Gastric Cancer

The high risk of recurrence after surgery for GC has led to a search for relapse prevention strategies to improve survival. Based on accumulating evidence, different adjuvant therapy options, including perioperative (neoadjuvant) and postoperative adjuvant chemotherapy, are available for localized GC. The pivotal randomized controlled clinical trials of perioperative (neoadjuvant) and adjuvant therapy for GC are listed in Table 4. Adjuvant (postoperative) systemic chemotherapy is typically used in Asian countries because D2 lymph node dissection is routinely performed in advanced GC [78]; perioperative chemotherapy (neoadjuvant plus adjuvant therapy) is mainly used in European countries [79], and adjuvant chemoradiation is historically preferred in North America [80]. These marked disparities between the East and West are attributable to surgical procedures and their outcomes, which result in considerable geographical variation in the preferred adjuvant treatment for localized GC [81,82].

### 5.1. Postoperative Adjuvant Therapy

Regarding postoperative adjuvant chemotherapy, the CLASSIC phase III trial showed that XELOX prolonged OS and disease-free survival (DFS) after D2 lymph node dissection in stage II–IIIB GC [83]. The ACTS-GC trial also revealed that S-1 chemotherapy for one year after D2 lymph node dissection improved OS in stage II or III GC [84]. The JACCRO GC-07 trial demonstrated that S-1 plus docetaxel therapy was more effective than S-1 monotherapy for relapse-free survival (RFS) in stage III GC [85]. Therefore, XELOX or S-1 is recommended as the postoperative adjuvant chemotherapy for stage II or III GC, while S-1 plus docetaxel is recommended for stage III GC in patients with adequate D2 dissection in Japan.

With regard to adjuvant CRT, in the INT-0116 trial, patients who received CRT after R0 resection had prolonged OS compared with those who received surgery alone [86]. However, more than 50% of the patients had lymph node dissection less than D1. The phase III randomized ARTIST 2 trial revealed no survival benefit of postoperative CRT after D2 dissection in node-positive GC [87]. In the phase III randomized CRITICS trial, patients who received appropriate preoperative chemotherapy and surgery did not display a survival benefit with the addition of postoperative radiation therapy [88,89,90].

### 5.2. Neoadjuvant and Perioperative Chemotherapy

With respect to perioperative treatment, several randomized trials have demonstrated the benefits of perioperative chemotherapy. In Europe, the phase III MAGIC trial revealed better PFS and OS for epirubicin, cisplatin, and fluorouracil (ECF) chemotherapy before and after surgery than for surgery alone [91].

In the FNCLCC/FFCD trial, perioperative chemotherapy with fluorouracil and cisplatin increased the curative resection rate, DFS, and OS [92]. Although this study was completed early owing to low accrual, fluorouracil and cisplatin may also be good options. Furthermore, a randomized controlled phase II/III FLOT-4 trial revealed that FLOT was better than epirubicin, cisplatin, and fluorouracil or capecitabine perioperative chemotherapy regimens [93]. The greatest benefit from perioperative chemotherapy may be derived from preoperative neoadjuvant chemotherapy because even in the FLOT4 trial, less than half of the study population completed the postoperative treatment as outlined in the protocol [94,95,96].

## 6. Prospects for Novel Therapies for Advanced Gastric Cancer

Although many molecular-targeted agents have been developed to treat GC, no agent, except trastuzumab, has shown efficacy as a first-line therapy [53]. However, advances in genomic analysis have revealed genetic subgroups that are attractive therapeutic targets [13]. Here, we provide an overview of the development of zolbetuximab for claudin 18.2 (CLDNl8.2) and bemarituzumab for fibroblast growth factor receptor 2b (FGFR2b), which are currently garnering remarkable attention as therapeutic targets.

CLDN18.2 is a transmembrane protein that forms tight junctions between cells and is expressed in many types of cancer cells, including gastric adenocarcinoma. In a randomized phase II trial (FAST) [97], zolbetuximab, an IgG1 antibody targeting CLDN18.2, in combination with standard therapy (epirubicin + oxaliplatin + capecitabine (EOX)), significantly improved OS (HR: 0.55, 95% CI: 0.39–0.77, *p* < 0.0005) and PFS (HR: 0.44, 95% CI: 0.29–0.67, *p* < 0.0005). Based on these results, a phase III study (the SPOT-LIGHT study) is currently ongoing. 

FGFR2, a receptor-type tyrosine kinase, is known to be one of the poor prognostic factors for GC. The findings of the FIGHT study, a randomized phase II trial evaluating the add-on effect of bemarituzumab to chemotherapy (FOLFOX) in the first-line treatment of FGFR2-positive advanced GC, were reported at ASCO 2021 [98]. The primary endpoint of PFS (9.5 vs. 7.4 months, HR: 0.68, 95% CI: 0.44–1.04) and the secondary endpoint of OS (19.2 vs. 13.5 months, HR: 0.60, 95% CI: 0.38–0.94) were significantly prolonged, and the response rates were 53% vs. 40%, respectively. Notably, in patients with FGFR2b expression in more than 10% of immunohistochemistry patients, the OS was 25.4 months vs. 11.1 months (HR: 0.41, 95% CI: 0.23–0.74), indicating a trend of an association between the overexpression of FGFR2b and treatment response.

## 7. Conclusions

In this review, we aimed to provide an overview of the recent advances in chemotherapy for patients with advanced GC. Although systemic therapy for GC has gradually improved OS over the past several decades, substantial challenges remain for oncologists to achieve ideal outcomes. As GC is a histologically, molecularly, and immunologically heterogeneous disease [99], biomarker-targeted therapy has received remarkable attention in recent years. Such marked attention is because the development of biomarkers based on an in-depth understanding of tumor molecular biology is expected to provide better patient stratification and selection and lead to patients benefitting from specific targeted therapies in GC. 

Currently, trastuzumab is the standard choice for advanced GC patients with HER2 overexpression based on the positive results of HER2-targeted therapies in patients with advanced GC. However, HER2 expression in GC is less than 20%, and the presence of tumor heterogeneity and resistance to trastuzumab are major limitations. To overcome these challenges, new therapeutic agents under investigation, such as antibody–drug conjugates and HER2-targeted bispecific antibodies, are expected. 

More recently, targeted therapy against PD-L1 has led to immunotherapy as a frontline treatment for advanced GC, following the results of CheckMate 649 and KEYNOTE-811. The use of immunotherapy is expected to increase in combination with other promising targeted agents. Patient selection using PD-L1 scores in ICI therapy has been investigated; however, the threshold for PD-L1 positivity remains unclear. Establishing biomarkers for immunotherapy is expected to optimize patient responses by improving patient selection for ICI treatment. In the future, a better understanding of the molecular characterization of GCs will likely aid the use of the best targeted and ICI therapies in relation to surgery, chemotherapy, and radiotherapy for GC.

## Figures and Tables

**Table 1:** Pivotal randomized controlled clinical trial for first-line human epidermal growth factor receptor 2 (HER2)-negative gastric cancer.

Study	N	Publication Year	Area	Treatment	Median OS (Month)	HR
JCOG9912	234	2009	Japan	5-FU	10.8	
	236			CPT-11 + CDDP	12.3	0.85
	234			S-1	11.4	0.83
SPIRITS	150	2008	Japan	S-1	11	0.77
	148			SP	13	
G-SOX	324	2015	Japan	SP	13.1	0.96
	318			SOX	14.1	
REAL-2	263	2008	UK and Australia	ECF	9.9	
	250			ECX	9.9	
	245			EOF	9.3	
	244			EOX	11.2	
ATTRACTION-4	362	2022	Japan, South Korea, and Taiwan	SOX/CapeOX	17.15	0.9
	362			SOX/CapeOX + nivolumab	17.45	
CheckMate649	482	2022	Global (29 countries)	CapeOX/FOLFOX	11.1	
	473			CapeOX/FOLFOX + nivolumab	14.4	0.7
	234			Nivolumab + ipilimumab	11.2	0.89

SP: S-1 and cisplatin; ECF: epirubicin, cisplatin, and 5-fluorouracil; ECX: epirubicin, cisplatin, and capecitabine; EOF: epirubicin, oxaliplatin, and 5-fluorouracil; EOX: epirubicin, oxaliplatin, and capecitabine; XELOX/capeOX: capecitabine and oxaliplatin; XP: capecitabine and cisplatin; SOX: S-1 and oxaliplatin; FOLFOX: fluorouracil, leucovorin, and oxaliplatin; HR: hazard ratio; OS: overall survival.

**Table 2:** Pivotal randomized controlled clinical trial for first-line human epidermal growth factor receptor 2 (HER2)-positive gastric cancer.

Study	N	Publication Year	Area	Treatment	Outcomes	HR
ToGA	234	2010	Global (24 countries)	FP/XP	Median OS: 11.1month	0.74
	236			FP/XP + trastuzumab	Median OS: 13.8 month	
KEYNOTE-811	131	2021	Global (20 countries)	FP/XELOX + trastuzumab	Objective response: 51.9%	
	133			FP/XELOX + trastuzumab + pembrolizumab	# Objective response: 74.4%	

FP: 5-fluorouracil and cisplatin; XELOX: capecitabine and oxaliplatin; XP: capecitabine and cisplatin; HR: hazard ratio; OS: overall survival. # Significant (22.7%) improvement in objective response rate in the pembrolizumab group (95% CI, 11.2–33.7; *p* = 0.00006).

**Table 3:** Pivotal randomized controlled clinical trial for second-line and subsequent chemotherapy for gastric cancer.

Study	N	Publication Year	Area	Treatment	Median OS (Month)	HR
REGARD	117	2014	Global (29 countries)	Placebo	3.8	0.776
	238			Ramucirumab	5.2	
RAINBOW	330	2014	Global (27 countries)	Paclitaxel	7.4	0.807
	335			Paclitaxel + ramucirumab	9.6	
ATTRACTION-2	324	2017	Japan, South Korea, and Taiwan	Placebo	4.14	0.63
	318			Nivolumab	5.26	
TAGS	170	2018	global (17 countries)	Placebo	3.6	0.69
	337			FTD/TPl	5.7	
DESTINY-Gastric 01	62	2020	Japan and South Korea	Physician’s choice of irinotecan or paclitaxel	8.4	0.59
	125			Trastuzumab deruxtecan	12.5	

FTD/TPI: Trifluridine/tipiracil; HR: hazard ratio; OS: overall survival.

**Table 4:** Pivotal randomized controlled clinical trial of perioperative (neoadjuvant) and adjuvant therapy for gastric cancer.

Study	N	Publication Year	Area	Localization of the Tumor	Recommended Resection	Treatment	Survival	HR
ACTS-GC	530	2011	Japan	Gastric 100%	D2	Surgery only	5-year OS: 61%	0.669
	529					S-1(1 year)	5-year OS: 72%	
CLASSIC	515	2014	South Korea	Gastric 98%, GEJ 2%	D2	Surgery only	5-year OS: 69%	0.6
	520					XELOX (8 cycles)	5-year OS: 78%	
JACCRO GC-07	459	2019	Japan	Gastric 100%	D2	S-1 (1 year)	3-year RFS: 50%	0.632
	454					S-1 plus docetaxel (1 year)	3-year RFS: 66%	
Intergroup 0116	277	2001, 2012	USA	Gastric 80%, GEJ 20%	D2	Surgery only	5-year OS: 28%	1.32
	282					Adjuvant CRT (45Gy + 5FU)	5-year OS: 43%	
ARTIST-2	182	2021	South Korea	Gastric 100%	D2	Adjuvant chemotherapy (S-1 for 1 year)	3-year DFS: 64.8%	
	181					Adjuvant chemotherapy (SOX for 6 month)	3-year DFS: 74.3%	0.692
	183					Adjuvant CRT (SOX plus RT 45 Gy)	3-year DFS: 72.8%	0.724
CRITICS	393	2018	The Netherlands, Sweden, and Denmark	Stomach 25%, GEJ 64%	at least a D1+	Perioperative chemotherapy (3 preoperative and 3 postoperative cycles of modified ECF)	Median OS: 43 months	1.01
	395					Preoperative chemotherapy with postoperative CRT (Capecitabine and cisplatin with concurrent RT 45 Gy)	Median OS: 37 months	
MAGIC	250	200	UK	Gastric 74%, lower esophageal/GEJ 26%	Undefined	Surgery only	5-year OS: 23%	0.75
	253					Perioperative chemotherapy (3 cycles of preoperative ECF and 3 cycles of postoperative ECF)	5-year OS: 36%	
FNCLCC/FFCD	111	2011	France	Stomach 25%, lower esophageal 11%, GEJ 64%	D2	Surgery only	5-year OS: 24%	0.69
	113					Perioperative chemotherapy (2–3 cycles of preoperative CF and 3–4 cycles of postoperative CF)	5-year OS: 38%	
FLOT4	356	2019	Germany	Stomach 44%, GEJ 56%	D2	3 preoperative and postoperative cycles of ECF/ECX	Median OS: 50 months	0.77
	360					4 preoperative and postoperative cycles of FLOT	Median OS: 30 months	

ECF: epirubicin, cisplatin, 5-fluorouracil: GEJ: gastroesophageal junction; XELOX: capecitabine and oxaliplatin; XP: capecitabine and cisplatin; SOX: S-1 and oxaliplatin; FLOT: fluorouracil, leucovorin, oxaliplatin, and docetaxel; XRT: radiotherapy with concomitant capecitabine; AC: adenocarcinoma; CRT: chemoradiotherapy; HR: hazard ratio; OS: overall survival; RFS: relapse-free survival.

## Data Availability

The data that support the findings of this study are available from the corresponding author upon reasonable request.
